# Invisible scars and trauma: The psychological cost of youth-led protests in Nepal and the global imperative for trauma‑responsive mental-health systems

**DOI:** 10.1371/journal.pmen.0000522

**Published:** 2026-01-27

**Authors:** Shishir Paudel, Anisha Chalise, Uday Narayan Yadav

**Affiliations:** 1 Kathmandu Institute of Child Health, Hepali Height, Kathmandu, Nepal; 2 Independent researcher, Kathmandu, Nepal; 3 National Centre for Epidemiology and Population Health, The Australian National University, Canberra, Australia; 4 International Centre for Future Health System, University of New South Wales, Sydney, Australia; PLOS: Public Library of Science, UNITED KINGDOM OF GREAT BRITAIN AND NORTHERN IRELAND

## Nepal’s 2025 Gen‑Z movement

In September 2025, Nepal witnessed an unprecedented youth-led movement driven largely by Generation Z (aged 13–28 years), with even schoolchildren joining the protests, reflecting the frustrations of a generation. What began as peaceful demonstrations against a governance system that is perceived to lack accountability, widespread corruption, nepotism, high youth unemployment, and a temporary social-media ban (viewed as a threat to civic freedom) quickly escalated into chaos, with the excessive use of force against protesters [1]. Gen-Z protesters demanded broader political accountability and reforms to support youths in the country. However, following the killing of young protesters, the movement turned violent, with attacks on the judiciary, legislative buildings, and police administration, together with the burning of several commercial complexes, supermarkets, and even government buildings [2], marking the most destructive civil disturbance in Nepal. According to official and media reports, at least 53 people were killed, and 2,429 were injured on the first day of the protest. An estimated 34 deaths resulted from gunshot wounds by civil force, and victims included children as young as 12 years. Hospitals were overwhelmed, and several were targeted during the clashes, heightening fear among both civilians and health workers [1,3]. Reports of escaped prisoners further intensified insecurity among the public and security forces, adding to the escalating psychological burden [2]. The death toll later reached 72 as bodies were recovered from government offices, houses, and other buildings that were set on fire. The unrest disrupted economic activities, caused extensive property damage, eroded public confidence in state institutions, and left many people without livelihoods [4].

## Gaps in crisis response and mental-health governance

The Government of Nepal announced financial compensation and established “Gen-Z clinics”, allocating NPR 4.8 million (33,565 USD) to provide free medical treatment for those physically injured [3]. While commendable, these interventions address only visible wounds and leave psychosocial distress largely unaddressed among survivors, families, witnesses, and other vulnerable groups. The brutality witnessed in person and repeatedly replayed through social and news media has left many reliving scenes of bloodshed, detention, and fear [1]. However, Nepal’s post-protest response has remained largely biomedical, focusing on physical injuries while overlooking enduring mental-health consequences that may persist for years. This absence of integrated, trauma-responsive care and mental-health preparedness within national emergency and conflict-response systems represents a substantial policy and practice gap.

Post-traumatic stress disorder (PTSD), depression, and anxiety are well-documented outcomes of exposure to violence, conflict, and disaster. Evidence shows that without early psychosocial intervention, affected children often develop persistent, clinically significant trauma-related disorders [5,6]. Such conditions often persist into adulthood, undermining educational attainment, relationships, and long-term productivity. Nepal, which already faces critical shortages of child and adolescent mental-health services [7], now risks facing an unrecognised epidemic of trauma-related disorders if timely actions are not taken. Considering the country’s limited resources and shortage of skilled human resource for mental health, the national capacity to respond to cumulative psychological stressors arising from continuous political unrest remains severely constrained. The Gen-Z movement exposes deep vulnerabilities in Nepal’s health governance and crisis-response mechanisms. Without sustained psychosocial initiatives, unresolved trauma may exacerbate societal instability as the country approaches the 2026 general election.

## Global pattern of youth political trauma

The Gen-Z movement is not unique to Nepal. Youth-led protests have emerged across countries, from Bangladesh and Indonesia to Peru and Morocco, where young protesters have faced state violence and detention [8,9]. However, there is limited evidence that governments have systematically implemented trauma‑informed mental‑health interventions targeting protest‑affected youth. In Bangladesh, high rates of PTSD and depression have been documented among youth affected by political violence, but there is no clear report of a dedicated government program to provide long-term protest-specific psychosocial care [10]. In conflict-affected countries such as Colombia, research has examined the resources and agency of young people to access support for mental health and emotional well-being, and pilot mental-health interventions have been implemented for young adults in post-conflict regions [11,12]. Nevertheless, as of now, there is no documented government program providing a structured, trauma‑informed, or psychosocial support scheme specifically for youth protestors in any of the countries experiencing Gen-Z movements.

These shared experiences underscore the need to integrate mental-health preparedness and trauma care into national crisis responses worldwide. These dynamics expose young people to direct and indirect trauma, yet mental-health preparedness remains insufficient in most national crisis frameworks. For South and Southeast Asian countries, where half the population is under 30, the psychological impact of political unrest represents an under-recognised public health and policy challenge. The Gen-Z movement in Nepal, therefore, reflects a broader global imperative. International frameworks, including the United Nations Convention on the Rights of the Child and the World Health Organization(WHO)‘s Comprehensive Mental Health Action Plan 2020–2030, oblige states to prioritise the mental well-being of young people during crises and recovery efforts [13,14].Global partners such as the WHO, United Nations Children’s Fund, and regional donors should support Nepal in developing trauma-responsive systems that extend beyond the hospital to communities.

A coordinated, multi-level mental-health response is urgently required. To date, no structured national initiative has been implemented to address the psychosocial and trauma-related impacts of the protests, leaving affected youths and families without formal psychological care or community-based support. Screening for PTSD, anxiety, and depression should be integrated into the outreach activities of Gen-Z clinics and primary health-care centres. Psychological first aid and trauma-informed counselling must be expanded through community health workers, teachers, and trained youth volunteers, employing evidence-based approaches. Dedicated funding for mental-health support should parallel physical injury compensation. Educational institutions can further contribute by incorporating structured resilience-building programmes that encourage open discussion of trauma, healing strategies, and reduce stigma.

## Conclusion

The political climate in Nepal remains fragile, and the potential for renewed unrest highlights the urgent need to strengthen mental-health preparedness. Youth exposed to violence during the Gen-Z movement may carry unresolved trauma that threatens civic participation, trust in institutions, and social cohesion. Failure to address these psychological impacts risks deepening social and health inequalities and undermining democratic stability, a concern relevant not only to Nepal but also to countries where mental health is neglected during civil unrest. As the individuals’ wounds and the country’s economy may heal, untreated psychological trauma may shape the collective mental health and social cohesion of an entire generation. Structured, trauma-responsive interventions, including early identification, psychosocial support, and resilience-building programmes, are essential to prevent long-term psychological harm. Recognising, preventing, and treating PTSD and other mental stress among Nepal’s youth is not only a clinical necessity but also a moral and political responsibility. The cost of neglect will resonate for decades.
